# Ayahuasca Beverages: Phytochemical Analysis and Biological Properties

**DOI:** 10.3390/antibiotics9110731

**Published:** 2020-10-24

**Authors:** Joana Gonçalves, Ângelo Luís, Ana Gradillas, Antonia García, José Restolho, Nicolás Fernández, Fernanda Domingues, Eugenia Gallardo, Ana Paula Duarte

**Affiliations:** 1 Centro de Investigação em Ciências da Saúde (CICS-UBI), Universidade da Beira Interior, Av. Infante D. Henrique, 6200-506 Covilhã, Portugal; joanadgoncalves13@gmail.com (J.G.); jose.restolho@ubi.pt (J.R.); fdomingues@ubi.pt (F.D.); apcd@ubi.pt (A.P.D.); 2 Laboratório de Fármaco-Toxicologia, UBI Medical, Universidade da Beira Interior, Estrada Municipal 506, 6200-284 Covilhã, Portugal; 3 CEMBIO, Center for Metabolomics and Bioanalysis, Facultad de Farmacia, Universidad San Pablo CEU, CEU Universities, Campus Monteprincipe, Boadilla del Monte, 28668 Madrid, Spain; gradini@ceu.es (A.G.); antogar@ceu.es (A.G.); 4 Cátedra de Toxicología y Química Legal, Laboratorio de Asesoramiento Toxicológico Analítico (CENATOXA), Facultad de Farmacia y Bioquímica, Universidad de Buenos Aires, Junín 956, C1113AAD Buenos Aires, Argentina; nfernandez@ffyb.uba.ar

**Keywords:** Ayahuasca, phytochemical characterization, antioxidant activity, anti-inflammatory activity, antimicrobial properties

## Abstract

Ayahuasca is a psychoactive beverage, originally consumed by indigenous Amazon tribes, of which consumption has been increasing worldwide. The aim of this study was to evaluate the phytochemical profile, as well as the antioxidant, anti-inflammatory and antimicrobial properties of decoctions of four individual plants, a commercial mixture and four mixtures of two individual plants used in the Ayahuasca preparation. For this purpose, a phytochemical characterization was performed, determining the content of flavonoids, total phenolic compounds, and analyzing the phenolic profile. Besides, 48 secondary metabolites were investigated by ultra-high performance liquid chromatography-quadrupole time-of-flight mass spectrometry (UHPLC-Q/TOF-MS) and their concentration estimated with real standards when present. The antioxidant activity was evaluated by both the β-carotene bleaching test and DPPH free radical scavenging assay, and the anti-inflammatory activity was determined by a protein denaturation method. Finally, the antimicrobial properties were evaluated using the disc diffusion assay, resazurin microtiter method, anti-quorum sensing and anti-biofilm activity assays. The obtained results showed that, in general, the samples have a high content of phenolic compounds and flavonoids with noticeable differences, reflecting on remarkable antioxidant and anti-inflammatory activities. Significant antimicrobial properties were also observed, with emphasis on the effect of *B. caapi* and *P. harmala* on planktonic and biofilm cells of *A. baumannii*, inhibiting both the biofilm formation and the production of violacein pigment.

## 1. Introduction

Ayahuasca is a psychoactive beverage that has been consumed for centuries [1,2]. Originally, it was used by indigenous tribes in the Northwest Amazon for therapeutic purposes and divine rituals; however, it has now expanded, being consumed worldwide [3,4,5]. The term “Ayahuasca” has Quechua origin and derives from the words “aya”, which means “spirit”, and “huasca”, meaning “vine” [1,2,4,5,6]. The significance of this beverage, also known as *yajé*, *daime*, *vegetal*, *capi*, *nate*, *hoasca*, *natema*, can be translated as “vine of the dead” or “vine of the soul” [2,4,7,8]. Traditionally, Ayahuasca consisted of a decoction prepared with the leaves of the *Psychotria viridis* bush and scraps of the stem of the *Banisteriopsis caapi* vine. However, over the years, several variations of this decoction have been developed [4,5]. Several analogues that can replace *P. viridis* are also known, namely *Psychotria carthagenesis*, *Brugmansia suaveolens*, *Nicotiana tabacum*, *Brunfelsia, spp*., *Daturaolens*, *Malouetia tamarquina*, *Tabernaemontana* spp., among others [1,9]. In the case of *B. caapi*, besides to analogs of natural origin (*Peganum harmala*), there are also synthetic analogs (tetrahydroharmine freebase/HCl, moclobemide and harmine freebase/HCl) that can replace the use of the traditional plant [1,3,10,11].

The Ayahuasca decoction contains β-carboline alkaloids such as harmine, tetrahydroharmine (THH) and harmaline, which come from *B. caapi*, and the hallucinogenic compound *N,N*-dimethyltryptamine (DMT) from *P. viridis* [12,13]. DMT is a serotonin 5-HT1A/2A/2C receptors agonist that, when ingested alone, is metabolized by peripheral monoamine oxidase-A (MAO-A), becoming harmless [14]. Nonetheless, when combined with the β-carboline alkaloids, can access the circulation and the central nervous system, since they temporarily inhibit MAO-A [12,14,15,16]. Additionally, THH also inhibits the reuptake of serotonin by increasing the effects of DMT [17]. This compound is hydrophobic and presents a low molecular weight, allowing it to quickly cross the blood-brain barrier. Furthermore, it is structurally similar to melatonin and serotonin, with a tryptamine moiety which provides high affinity for neurological receptors and triggers more robust behavioral responses [18].

In the last twenty-five years, the Ayahuasca consumption has expanded worldwide (United States of America, Europe, Africa and Asia) raising concerns about the possible negative effects of its consumption, but also curiosity about the potential therapeutic effects described [13,17,19,20].

The increase of the antimicrobial resistance by pathogens is of major concern, since it leads to an increase in morbidity and mortality, endangering public health [21,22]. Thus, antibiotics that were used against pathogens are no longer effective, namely against Gram-positive bacteria such as *Staphylococcus aureus* that developed resistance against methicillin [23,24]. Additionally, studies performed on Gram-negative bacteria, such as *Acinetobacter baumannii*, *Escherichia coli* and *Pseudomonas aeruginosa*, have shown the emergence of antibiotic resistance [25,26]. Thus, it is crucial to search for new compounds with antimicrobial properties that allow the treatment of infections by resistant pathogens [27]. Higher plants have classes of compounds that can be used as sources of antibiotics, so it is important to evaluate the antimicrobial effects of those compounds in order to identify new molecules with potential inhibitory properties of pathogenic microorganisms [27,28].

The present work describes the phytochemical characterization of four individual plants, a commercial mixture and four plant mixtures used in the preparation of the Ayahuasca decoctions. The antioxidant, anti-inflammatory and antimicrobial properties of those plant samples were also evaluated.

## 2. Results and Discussion

The search for new plant-derived compounds with bioactive properties is crucial. Thus, and considering the potential effects of Ayahuasca, in this work, the biological activities of the plant materials used in the preparation of this beverage, namely antioxidant, anti-inflammatory and antimicrobial properties, were evaluated. In this sense, four decoctions of Ayahuasca were prepared (with two different plant materials, one source of DMT and another of β-carboline alkaloids). Besides, individual decoctions of each plant used in the preparation of Ayahuasca were also prepared. In addition, a commercial mixture was also purchased and evaluated.

### 2.1. Phytochemical Characterization and Phenolic Profile

Ayahuasca has been shown to have important beneficial health effects [12,17,29,30,31,32,33,34,35]. Considering that there is a small number of phytochemical studies of the decoctions prepared from the plants *P. viridis*, *B. caapi*, *P. harmala* and *M. hostilis*, in the present study the determination of the content of flavonoids and total phenolics in these samples was performed. Additionally, the phenolic profile of the samples was determined by ultra-high performance liquid chromatography-quadrupole time-of-flight mass spectrometry (UHPLC/ESI-QTOF-MS).

Phenolic compounds are among the main secondary metabolites of plants, and it is possible to find some of them in all plants [36,37,38]. These compounds have some interesting properties from a clinical point of view [37,39]. In the present study, the total phenolics were determined using the Folin-Ciocalteu colorimetric method (Table 1). Although some studies describe limitations of the Folin-Ciocalteu colorimetric method, it remains widely used for the determination of total phenolics [40]. All samples showed substantial concentrations of total phenolics, with *M. hostilis*, commercial mixture and the mixture of *M. hostilis* and *B. caapi* showing the highest concentrations, and *P. harmala* the lowest content of total phenolics. Recently, Hadadi et al. [41], developed a study where determined the content of total phenols, using the same method, having also verified the presence of these compounds in extracts of *P. harmala*.

Flavonoids have beneficial biological activities, namely anti-inflammatory, antimicrobial, antioxidant, cytotoxic and anti-tumor activities [42,43]. In the present study, flavonoids were determined by the aluminum chloride colorimetric method (Table 1). All samples were found to have flavonoids in their composition, with *P. harmala* and the mixture of *P. viridis* and *P. harmala* the samples having the highest flavonoid content. Contrariwise, the samples of *B. caapi* and the mixture of *B. caapi* and *M. hostilis* showed the lowest levels of flavonoids.

**Figure A1:**
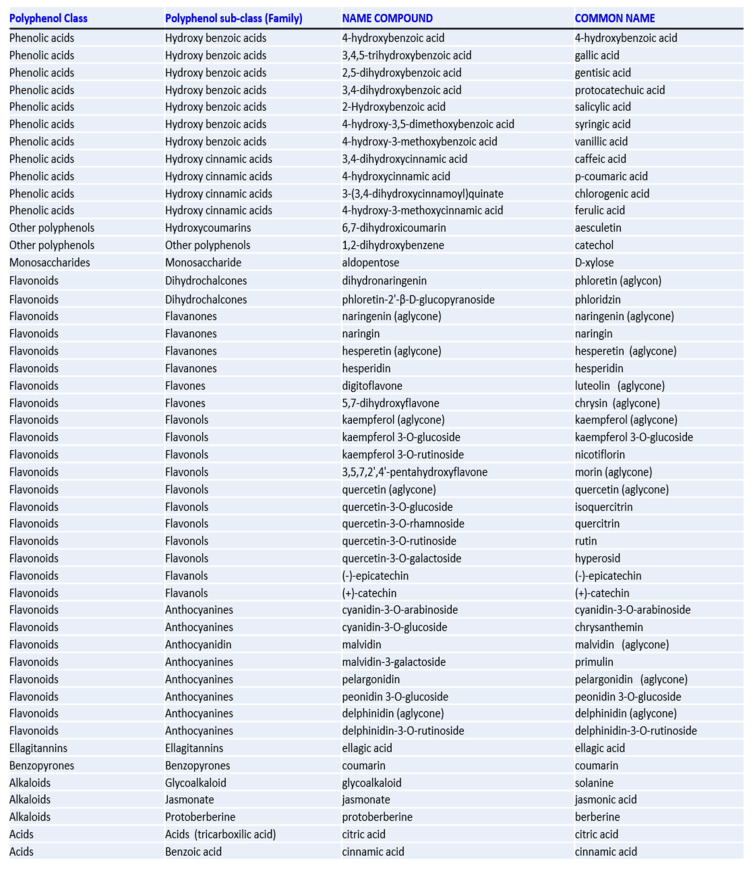
Library of phytochemical compounds used in the analysis by UHPLC-Q/TOF-MS.

The samples were then analyzed by liquid chromatography with high resolution mass spectrometric detection in order to identify the compounds and to further complement the initial phytochemical characterization. Thus, the identification of compounds was carried out by comparing their retention times and accurate mass spectra provided by the UHPLC-QTOF-MS with those of authentic standards when available. The phytochemical library of 48 standards (Appendix A Figure A1) was used to characterize the metabolites present in the methanolic extracts. Thus, it was possible to verify that two groups of compounds were mainly present: hydroxybenzoic acids and flavonoids. The concentration of the identified compounds was estimated by comparing their peak areas in the chromatograms from the plant extracts with those of the corresponding standard solutions freshly prepared and analyzed by duplicate in the same batch as samples. The results are shown in Table 2. In the sample of *P. viridis* it was possible to quantify two hydroxybenzoic acids (protocatechuic acid, 4-hydroxybenzoic acid), two hydroxycinnamic acids (chlorogenic acid and neochlorogenic acid) and six flavonoids ((+)-catechin, (-)-epicatechin, quercetin-3-O-galactoside, quercetin-3-O-glucoside, quercetin-3-O-rutinoside and kaempferol-3-O-rutinoside). These results are similar to those obtained by Ma et al. [44], where the phenolic compounds of a sample of *P. viridis* were analyzed by liquid chromatography coupled to mass spectrometry, being (+)-catechin and (-)-epicatechin detected. Additionally, five other compounds were also detected (gallic acid, (+)-gallocatechin, dihydromyricetin, (+)-catechin-3-O-gallate and myricitrin) that are not part of the library used in this work [44]. In the sample of *P. harmala* it was only possible to quantify the hydroxybenzoic acids, protocatechuic, gentisic and salicylic acids. The remaining compounds were either not detected, or are below the limit of quantification. Regarding the sample of *M. hostilis*, protocatechuic, 4-hydroxybenzoic and salicylic acids, and the flavonoids (+)-catechin and (-)-epicatechin were quantified. Regarding the *B. caapi* sample, the hydroxybenzoic acids, protocatechuic and salicylic, and the flavonoids (+)-catechin, (-)-epicatechin, quercetin-3-O-glucoside, quercetin-3-O-rutinoside and phlorizin, were quantified. Finally, the commercial mixture was also analyzed by the same analytical method, allowing the quantification of three hydroxybenzoic acids (protocatechuic, 4-hydroxybenzoic and salicylic acids) and five flavonoids ((+)-catechin, (-)-epicatechin, quercetin-3-O-galactoside, quercetin-3-O-glucoside and quercetin-3-O-rutinoside). The chemical composition and the proportion of the identified compounds are variable in the different analyzed samples. It is difficult to compare the results obtained for the samples of *B. caapi*, *M. hostilis*, *P. harmala* and for the commercial mixture, since most chromatographic studies focus on the detection of psychoactive compounds, such as DMT, or β-carboline alkaloids [3,45,46,47,48,49,50]. Therefore, future research on these plant samples should be focused on the phytochemical characterization and potential bioactive effects associated with these compounds.

### 2.2. Antioxidant Activity

In this study, the antioxidant activity of the Ayahuasca decoctions was evaluated in order to identify new sources of antioxidants. Initially, the antioxidant activity of the extracts was evaluated by the 2,2-diphenyl-1-picrylhydrazyl (DPPH) free radical scavenging assay and the results are presented in Table 3. This colorimetric assay is widely used because it is quick and easy, consisting in the evaluation of the potential for free radical scavenging of the samples [51]. Observing the results, it is possible to verify that the samples of *M. hostilis*, the mixtures of *M. hostilis* and *B. caapi* and *M. hostilis* and *P. harmala*, and the commercial mixture showed a “very strong” antioxidant activity, because their antioxidant activity index (AAI) values were higher than 2.0 [52]. Otherwise, the sample of *P. harmala* presented values of AAI below 0.5, and, therefore, “poor” antioxidant activity [52]. The remaining samples showed “strong” antioxidant activity [52]. These results may be related with the phenolic compounds present in the samples, given that the ones with the better antioxidant activity (mixture of *M. hostilis* and *B. caapi* and a commercial mixture) are also those with higher concentration of total phenolics (Table 1). To the best of our knowledge, there are no previous studies where the antioxidant activity of *P. viridis*, *B. caapi* and *M. hostilis* was evaluated. Regarding the results obtained for *P. harmala*, these are very similar to those obtained by other researchers, where the IC_50_ values are always greater than 100, resulting in reduced antioxidant activity [53,54].

The comparison of the results of the antioxidant activity of a determined sample is not linear, since the mechanism of action is very complex and varies within matrices. Additionally, the measurement of this activity depends on the method employed [27]. For these reasons, and in order to better understand the antioxidant mechanism of Ayahuasca decoctions, their antioxidant activity was also evaluated by the β-carotene bleaching test (Table 3). This assay is widely used in the evaluation of the antioxidant activity of samples of natural origin, since it allows the evaluation of the ability of the samples to inhibit the lipid peroxidation [40,55]. In this test, the antioxidant activity evaluation is undertaken by comparing two competitive chemical reactions involving the potentially antioxidant compounds present in the samples and the antioxidant model β-carotene [56]. As it was observed in the DPPH assay, the *P. harmala* sample showed the highest IC_50_ value and, consequently, the lowest antioxidant activity. Contrariwise, the samples with lower IC_50_, and, therefore, greater antioxidant activity, were the commercial mixture and *P. viridis*.

In general, the results obtained allowed concluding that the Ayahuasca is a good source of bioactive compounds with the ability to scavenge free radicals and to inhibit the lipid peroxidation.

### 2.3. Anti-Inflammatory Activity

Protein denaturation is an important indicator of an inflammation process, since it occurs during tissue damage, leading to the production of auto-antigens [57,58]. Thus, in this work, the anti-inflammatory activity of the samples was studied, by assessing its capacity to inhibit protein denaturation. Although this method is not a direct test, it is often used to assess the anti-inflammatory potential of plant samples [59]. Observing the results (Table 4) it is possible to verify that the samples with the highest IC_50_ and, consequently, with the lowest anti-inflammatory activity are *P. viridis* and *B. caapi*. The samples with the best anti-inflammatory activity are *P. harmala*, *M. hostilis*, the mixture of *M. hostilis* and *P. harmala* and the commercial mixture. The results obtained for *P. harmala* are in agreement with the literature, since previous studies corroborate its anti-inflammatory potential [60,61]. However, for the remaining samples, no studies were found to compare the results.

### 2.4. Antimicrobial Activity

Currently, the resistance to conventional drugs by pathogenic microorganisms is a major concern, and the search for alternatives of natural origin has been growing [62]. Thus, in this work, the potential antimicrobial activity of the Ayahuasca decoctions was evaluated against four Gram-positive and four Gram-negative bacteria. The tested strains were chosen because they are human infective, being some of them well known for their pathogenicity and resistance to antibiotics. Namely, *L. monocytogenes* and *B. cereus*, known foodborne pathogens, and *E. faecalis*, *S. aureus*, *E. coli*, *A. baumannii*, *P. aeruginosa* and *S.* Typhimurium, responsible for several health-related infections [63,64,65,66,67,68,69,70].

Initially, the disc diffusion assay was performed, with some samples presenting antibacterial activity (Table 5). Analyzing the results, it was verified that six samples inhibited the bacterial growth in all strains. However, the commercial mixture was unable to inhibit the growth of *E. faecalis*, and slightly inhibited the growth of *L. monocytogenes* and *S.* Typhimurium. Additionally, the mixture of *P. viridis* and *B. caapi* was not able to inhibit the growth of *L. monocytogenes*, *S.* Typhimurium, *E. coli* and *E. faecalis*. Regarding the mixture of *P. viridis* and *P. harmala*, there was also a reduced inhibition in the growth of *L. monocytogenes* and absence of growth inhibition of *E. faecalis*. The other samples showed remarkable antibacterial activity in all the tested strains. The strain that was less susceptible to the samples was *E. faecalis*, with a range of inhibition diameters between 6.00 mm and 10.13 mm. These results can be explained by the ability of this microorganism to adapt to severe situations, namely environmental changes, salt concentrations, extreme alkaline pH, or even to the deprivation of nutrition [71]. Otherwise, the most promising results were observed for *S. aureus* and *A. baumannii*, with inhibition diameters ranging between 20.39 mm and 13.27 mm and between 17.81 mm and 11.04 mm, respectively. It is also important to note that in this study it was possible to observe antimicrobial activity against Gram-negative bacteria, which usually have greater resistance to samples of natural origin [72,73].

After the initial screening of the antimicrobial potential of the samples, the minimum inhibitory concentration (MIC) values were determined. For this, the resazurin microtiter assay was performed (Table 6). The strains that presented the lowest MIC and, therefore, were more susceptible to the action of the samples, were *B. cereus* and *A. baumannii*, with values between 0.156 mg/mL and 5 mg/mL and between 0.625 mg/mL and 5 mg/mL, respectively. Similarly to what was verified in the disc diffusion assay, the mixtures of *P. viridis* and *B. caapi* and *P. viridis* and *P. harmala* showed the least promising results, with MIC values varying between 2.5 mg/mL and >10 mg/mL. However, these samples and the commercial mixture showed considerable MIC values against strains where no inhibition was observed in the disc diffusion assay. This result may be related to the poor diffusion of the extracts in the agar plates [27]. The samples that, in general, showed better MIC values, and consequently greater antimicrobial activity, were the ones of *B. caapi* and *P. harmala*. In previous studies, the antimicrobial action of *P. harmala* extracts against *E. coli* and *S.* Typhimurium was already reported [74]; however, the MIC values obtained in that study (0.625 mg/mL) were lower than those obtained now (1.25 mg/mL for *E. coli* and 2.5 mg/mL for *S.* Typhimurium). Bussmann et al. [75], also determined the MIC for *B. caapi* against *S. aureus* and *E. coli*, but the values presented in that study (0.0625 mg/mL for *E. coli* and 1 mg/mL for *S. aureus*) are lower than those now determined. Nevertheless, these comparisons must be made with caution, since in the present study the samples consists of a decoction, whereas in the previous studies, methanolic [74], and ethanolic [75], extracts were used, which allows a better extraction of potential bioactive compounds. Furthermore, the differences in susceptibility of the used strains would also affect the results.

Considering the antimicrobial activity demonstrated by the samples, their anti-quorum sensing properties were also evaluated (Table 7). For that, the biomonitor strain *Chromobacterium violaceum* ATCC 12472 was used, which produces the pigment violacein and uses signal molecules of N-acyl homoserine lactone in order to monitor population density [59]. Analyzing the results, it was observed that, with the exception of *M. hostilis*, all samples were able to inhibit the production of violacein and, consequently, the quorum sensing. However, the samples of *B. caapi* and *P. harmala* stand out, as they produced a diameter of inhibition violacein production much higher than the other samples (13.26 mm and 13.16 mm, respectively). It should be noted that the inhibition diameters of these two samples were greater than that of resveratrol, used as a positive control.

Over the years, the antimicrobial properties of some phenolic compounds, namely protocatechuic acid [76], gentisic acid [77], catechin and epicatechin [78], and other flavonoids [36,79,80,81,82,83] were reported. A high amount of these phenolic compounds in plants can lead to a more effective response in defense against pathogens [36,79,80,81,82,83]. Analyzing the Table 1, it is possible to verify that *P. harmala* presents the highest flavonoid content. Thus, and taking into account the antimicrobial activity previously described for flavonoids, the promising results obtained with this sample may be related to this group of compounds. Similarly, the results obtained for *B. caapi* can also be related to its phytochemical composition. Observing the Table 2, it is possible to verify that *B. caapi* presents considerable values of protocatechuic acid, catechin and epicatechin. As previously mentioned, the antimicrobial activity of catechin and epicatechin has been described, and their action against pathogens is known.

**Figure 1:**
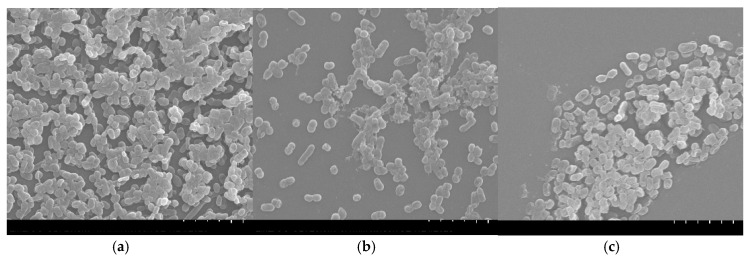
Scanning electron microscopy (SEM) images of *Acinetobacter baumannii* biofilms formed in the presence of two samples of Ayahuasca decoctions: (**a**) *A. baumannii* LMG 1025 biofilm (control); (**b**) *A. baumannii* LMG 1025 biofilm formed in the presence of *B. caapi* (0.5 × MIC); (**c**) *A. baumannii* LMG 1025 biofilm formed in the presence of *P. harmala* (0.5 × MIC). MIC—minimum inhibitory concentration; magnification 5000×.

*A. baumannii* is a pathogen responsible for multidrug-resistant nosocomial infections [84]. This microorganism is often associated with bloodstream infections and pneumonia associated with ventilation, which can even be fatal [70,84]. Additionally, *A. baumannii* has a plastic genome, which allows adaptation to adverse and stressful environments [70]. The virulence factors of this pathogen are known, namely the ability to form biofilms [59]. Biofilms produced by bacteria are highly resistant, as they are protected by the extracellular matrix [85]. In addition, the lower susceptibility of Gram-negative bacteria to inhibition by plant extracts has been described [72,73]. Given the promising results of *B. caapi* and *P. harmala*, concerning the antibacterial and anti-quorum sensing activities, their anti-biofilm activity against *A. baumannii* was further evaluated. For that, biofilms formed in polystyrene coupons in the presence of the samples were observed by SEM (Figure 1). Analyzing the Figure 1a it is possible to verify that the biofilm of *A. baumannii* presents a three-dimensional structure with several layers of cells connected to each other. In contrast, when the biofilm was formed in the presence of *B. caapi*, the *A. baumannii* cells appear in small number, with no connection between them and without the three-dimensional structure (Figure 1b). Observing Figure 1c, it appears that the small number of *A. baumannii* cells are partially destroyed when the biofilm grew in the presence of *P. harmala*. These results suggest that *P. harmala* and *B. caapi* present anti-biofilm activity against *A. baumannii*. Together with the results obtained in the anti-quorum sensing test, these data allow to infer that these samples are able to inhibit biofilm formation, not only by inhibiting the bacterial growth, but also by inhibiting the cells adhesion to the surface [86].

## 3. Materials and Methods

### 3.1. Sample Preparation

The vegetal samples of *P. viridis*, *B. caapi*, *M. hostilis* and *P. harmala*, as well as the commercial mixture (without information about its composition) were acquired online from the Shayana Shop (https://www.shayanashop.com, Amsterdam, The Netherlands). The decoctions of Ayahuasca were prepared according to a recipe kindly provided by Dr. Nicolás Fernández (Facultad de Farmacia y Bioquímica, Universidad de Buenos Aires, Buenos Aires, Argentina). Five decoctions of each individual plant samples were prepared. For this purpose, 0.210 g of each vegetal sample were macerated in a mortar with a few drops of water. This preparation was transferred to a Schott flask and 250 mL of ultra-pure water were added. The Schott flasks were boiled at 100 °C for 4 h. Similarly, four decoctions were prepared where two of the above vegetal samples were mixed (*P. viridis* and *B. caapi*; *P. viridis* and *P. harmala*; *M. hostilis* and *B. caapi*; *M. hostilis* and *P. harmala*). Therefore, in each mixture there is a source of DMT and a source of β-carboline alkaloids, according to works previously developed by our research group [87]. Finally, the samples were cooled, filtered, frozen at −80 °C and freeze-dried.

### 3.2. Phytochemical Characterization and Phenolic Profile

#### 3.2.1. Total Phenolic Compounds Determination

The total phenolic compounds were determined by the Folin–Ciocalteu colorimetric method [27]. For that, the samples were dissolved in methanol (50 mg/mL) and, subsequently, 50 μL of these solutions, or gallic acid (standard phenolic compound), were mixed with 450 μL of distilled water. Then, 2.5 mL of 0.2 N Folin–Ciocalteu reagent were added, the samples were left to stand for 5 min and, after that time, 2 mL of Na_2_CO_3_ (75 g/L) were added. The reaction mixtures were incubated at 30 °C for 90 min. Subsequently, the total phenolic content was determined by colorimetry (765 nm), using a standard curve prepared with methanolic solutions of gallic acid (*y* = 0.001*x*; R^2^ = 0.9845). The tests were carried out in triplicate, and the results were expressed as gallic acid equivalents (mg GAE/g sample) [40].

#### 3.2.2. Flavonoids Determination

The flavonoid content was determined by the aluminum chloride colorimetric method, following a previously described methodology, using quercetin as standard [27]. Thus, to 500 μL of each methanolic sample (50 mg/mL), 1.5 mL of methanol, 100 μL of aluminum chloride (10%, w/v), 100 μL of potassium acetate (1 M) and 2.8 mL of distilled water were added. This mixture was incubated for 30 min at room temperature. Subsequently, the flavonoid content was determined by colorimetry (415 nm), using a calibration curve prepared with methanolic solutions of quercetin (*y* = 0.0146*x*; R^2^ = 0.9964). The tests were performed in triplicate and the results were expressed as quercetin equivalents (mg QE/g of sample).

#### 3.2.3. Determination of the Phytochemical Profile by UHPLC/ESI-QTOF-MS

The identification of secondary metabolites present in the vegetal samples was performed following a methodology previously developed by the Center of Metabolomics and Bioanalysis (CEMBIO) based on analysis by UHPLC/ESI-QTOF-MS [88,89]. For that, a methanolic extraction was performed as follows: 300 μL of methanol was added to 30 mg lyophilized powder sample. The mixture was vortexed for 2 min, sonicated for 15 min and centrifuged at 10,000× *g* for 5 min at 4 °C. The supernatants were then collected and transferred to a Chromacol vial (Thermo Fisher Scientific, Madrid, Spain) for LC/MS analysis. The whole procedure was performed by duplicate. Then, samples were analyzed on a 1290 Infinity series UHPLC system coupled with an electrospray ionization source (ESI) with Jet Stream technology to a 6545 iFunnel QTOF/MS system (Agilent Technologies, Waldbronn, Germany). For the separation, a volume of 2 μL was injected in a reversed-phase column (Zorbax Eclipse XDB-C18 4.6 × 50 mm, 1.8 µm, Agilent Technologies) at 40 °C. The flow rate was 0.5 mL/min with a mobile phase consisting of solvent A: 0.1% formic acid in ultrapure water, and solvent B: methanol. Gradient elution consisted of 2% B (0–6 min), 2–50% B (6–10 min), 50–95% B (11–18 min), 95% B for 2 min (18–20 min), and returned to starting conditions 2% B in one minute (20–21 min) to finally keep the re-equilibration with a total analysis time of 25 min. Detector was operated in full scan mode (*m*/*z* 50 to 1500), at a scan rate of 1 scan/s both in positive and negative ESI mode. Accurate mass measurement was assured through an automated calibrator delivery system that continuously introduced a reference solution, containing masses of *m*/*z* 121.0509 (protonated purine) and *m*/*z* 922.0098 (protonated HP-921) in positive ESI mode; whereas *m*/*z* 119.0363 (proton abstracted purine) and *m*/*z* 966.0007 (formate adduct of HP-921) in negative ESI mode. The capillary voltage was ± 4000 V for positive and negative ionization mode. The source temperature was 225 °C. The nebulizer and gas flow rate were 35 psig and 11 L/min respectively, fragmentor voltage to 75 V and a radiofrequency voltage in the octopole (OCT RF Vpp) of 750 V.

For the study, Mass Hunter Workstation Software LC/MS Data Acquisition version B.07.00 (Agilent Technologies) was used for control and acquisition of all data obtained with UHPLC/MS-QTOF.

### 3.3. Evaluation of Antioxidant Activity

#### 3.3.1. DPPH Scavenging Assay

The antioxidant activity of the samples was determined by the radical scavenging activity method using the 2,2-diphenyl-1-picryhydrazyl (DPPH) free radical [52]. Briefly, to the methanolic solutions of the samples at different concentrations (100 µL), three methanolic solutions of DPPH (3.9 mL) were added at concentrations of 0.2, 0.1242 and 0.08 mM. The control was prepared by mixing methanol (100 µL) with 3.9 mL of each DPPH solution. The reaction mixtures were incubated at room temperature for 90 min and in the absence of light. After that, the absorbances were measured at 517 nm. The radical scavenging activity was calculated using the following formula:

I%= ((Abs_0_ − Abs_1_)/Abs_0_) × 100 (1)

where Abs_0_ corresponds to the control absorbance and Abs_1_ was the absorbance in the presence of the test samples at different concentrations. The IC_50_ was calculated graphically, using a linear calibration curve, plotting the sample concentrations in relation to the corresponding percentage of inhibition. The antioxidant activity was expressed as the antioxidant activity index (AAI), calculated as follows: AAI = (final concentration of DPPH in the control sample)/(IC_50_) [52]. The AAI allowed to classify the antioxidant activity of the samples as: Poor (AAI < 0.5), moderate (0.5 < AAI ≤ 1.0), strong (1.0 < AAI < 2.0) or very strong (AAI ≥ 2.0) [52]. All tests were performed in duplicate and gallic acid was used as control.

#### 3.3.2. β-Carotene Bleaching Test

A solution of β-carotene was prepared by dissolving 20 mg in 1 mL of chloroform. To 500 µL of this solution, 180 µL of linoleic acid, 400 mg of Tween 40 and 1 mL of chloroform were added. Then, the chloroform was evaporated on a rotary vacuum evaporator for 5 min at 45 °C. Subsequently, 100 mL of distilled water saturated with oxygen were slowly added to the mixture, which was then vigorously stirred to form an emulsion. After that, 2.5 mL of this emulsion were mixed with 300 µL of the extracts in methanol at different concentrations. A control was prepared by adding 2.5 mL of emulsion to 300 µL of methanol. The tubes were shaken and placed in a water bath at 50 °C for 1 h. After this time, the absorbances of the samples were measured at 470 nm, using an emulsion without β-carotene as blank. The measurements were performed in triplicate at 0 h (initial time) and at 1 h (final time). The antioxidant activity was measured in terms of the percentage of inhibition of β-carotene oxidation by:

% Inhibition = (Abs_t_ = 1_sample_ − Abs_t_ = 1_control_)/(Abs_t_ = 0_control_ − Abs_t_ = 1_control_) (2)

where Abs_t = 1_ was the absorbance of the sample or control at the final incubation time and Abs_t = 0_ was the absorbance in the control at initial incubation time [27]. Butylated hydroxytoluene (BHT) was employed as standard antioxidant compound.

### 3.4. Anti-Inflammatory Activity

The determination of anti-inflammatory activity was performed by assessing the ability of the samples to inhibit protein denaturation [59]. Thus, a 1% (w/v) solution of bovine serum albumin (BSA) in phosphate buffer solution (PBS) was prepared, with the pH adjusted to 6.8 using glacial acetic acid. Then, 900 µL of this solution was added to 100 µL of the samples previously diluted in dimethyl sulfoxide (DMSO) in tubes preheated to 37 °C. Then, the tubes were incubated at 72 °C for 10 min and after that time they were placed on ice for 10 min. A control consisting of distilled water was prepared. Finally, absorbance measurements were performed in triplicate using a microplate reader (BIO-RAD, Hercules, CA, USA) at 620 nm. The percentage of inhibition of protein denaturation was calculated using the following equation:

%I = 100 – ((Abs_sample_ × 100)/(Abs_control_)) (3)

where Abs_control_ is the absorbance of the control and Abs_sample_ is the absorbance of each sample [59]. Acetylsalicylic acid was used as positive control.

### 3.5. Determination of Antimicrobial Activity

The antimicrobial activity of Ayahuasca decoctions was evaluated against four Gram-positive bacterial species (*Staphylococcus aureus* ATCC 25923, *Bacillus cereus* ATCC 11778, *Listeria monocytogenes* LMG 16779 and *Enterococcus faecalis* ATCC 29212) and four Gram-negative (*Acinetobacter baumannii* LMG 1025, *Pseudomonas aeruginosa* ATCC 27853, *Escherichia coli* ATCC 25922 and *Salmonella* Typhimurium ATCC 13311). Stock cultures were kept at 20% glycerol at −80 °C. All strains were subcultured 24 h before the antimicrobial tests on Brain Heart Infusion Agar (BHI-A) plates.

#### 3.5.1. Disc Diffusion Assay

The antimicrobial activity of each extract was determined using the disc diffusion assay following the M2-A8 method, described by Clinical Laboratory and Standards Institute (CLSI). The inoculum was prepared by suspending the bacterial species in sterile saline solution to 0.5 McFarland units (about 1 to 2 × 10^8^ colony-forming units/mL (CFU/mL)). Then, discs (6 mm in diameter) were impregnated with 15 μL of each sample (3 mg/disc). Negative controls were prepared with DMSO (15 μL/disc) and positive controls were prepared with tetracycline (30 μg/disc). The discs were placed on the inoculated agar plates, which were then incubated for 24 h at 37 °C. After this time, the inhibition zones were checked, and the diameters were measured using a digital pachymeter. All experiments were carried out in triplicate [90].

#### 3.5.2. Resazurin Microtiter Method

The values of the minimum inhibitory concentrations (MIC) of the samples were determined using the resazurin microtiter assay [86]. Initially, the extracts were prepared in Müeller–Hinton Broth (MHB) at a concentration of 20 mg/mL, for strains *E. faecalis* and *L. monocytogenes* the culture medium used was Tryptic Soy Broth (TSB). A 96-well plate was marked and a volume of 100 μL of sample was pipetted into the first row of the plate. To all other wells, 50 μL of MHB or TSB were added, and serial twofold dilutions were performed. Then, 10 μL of resazurin indicator solution (0.1% diluted in MHB or TSB) were added. After that, 30 μL of MHB or TSB were added and, finally, 10 μL of bacterial suspension (0.5 McFarland units) were added to each well. The tests were performed in triplicate and tetracycline was used as a positive control. The plates were incubated for 18 h at 37 °C. The color change to colorless or pink was evaluated visually and registered as positive, with MIC being considered as the lowest concentration where the color change occurred [86].

#### 3.5.3. Anti-Quorum Sensing Properties: Solid Diffusion Assay

The anti-quorum sensing properties were evaluated using the biomonitor strain *Chromobacterium violaceum* ATCC 12472. The bacterial suspension was obtained by overnight aerobic growth in Luria-Bertani (LB) culture medium (250 rpm, 30 °C). Then, the bacterial suspension was adjusted to an OD_620nm_ of 1 and subsequently seeded on LB agar plates. Subsequently, discs were impregnated with each sample (3 mg/disc), placed on the plates and incubated for 24 h at 37 °C. After the incubation period, the anti-quorum sensing properties were evaluated by inhibiting the production of the violacein pigment around the disc, and the inhibition diameters were measured using a digital pachymeter. DMSO (15 μL/disc) was used as a negative control and resveratrol (5 μg/disc) as a positive control. The tests were performed in triplicate [59].

#### 3.5.4. Anti-Biofilm Activity

The anti-biofilm activity of the *B. caapi* and *P. harmala* decoctions was evaluated by scanning electron microscopy (SEM) for *A. baumannii* LMG 1025. Initially, *A. baumannii* LMG 1025 was grown overnight in LB (250 rpm, 37 °C). The *A. baumannii* LMG 1025 biofilms were formed in polystyrene coupons placed in 12-well plates. Thus, 500 μL of the bacterial suspension (OD_600nm_ of 0.02) and 500 μL of the samples dissolved in LB at a concentration of 0.5× MIC were placed in each well. The growth control consisted in 500 μL of LB with 500 μL of the bacterial suspension. The plates were incubated for 24 h at 37 °C. Then, the biofilms were washed twice with sterile saline solution and fixed with 2.5% glutaraldehyde (v/v) for 4 h at 4 °C. Subsequently, the samples were washed with PBS and the dehydration was carried out in a series of ethanol for 20 min each (30, 50, 70, 80, 90% (v/v) and absolute). The samples were then left to dry overnight in a desiccator. Finally, the biofilms were coated with gold and observed by SEM of variable pressure (S-3400N; Hitachi, Tokyo, Japan), using a voltage of 20.0 kV, emission of 100.0 μA and magnification of 5000× [59,86].

## 4. Conclusions

This work allowed a more deepened knowledge of the phytochemical composition of samples of Ayahuasca and the plant species used in the preparation of this psychoactive beverage. The ability of the samples to inhibit the lipid peroxidation and the capability to scavenge free radicals were demonstrated, indicating the antioxidant properties of these samples. The anti-inflammatory properties by the ability to inhibit protein denaturation were also evaluated. In addition, the antimicrobial activity of the samples was tested, together with the anti-quorum sensing and anti-biofilm potential against *A. baumannii* of *B. caapi* and *P. harmala* samples. The whole set of results obtained allowed a better knowledge of Ayahuasca decoctions; however, future work is necessary to fully understand the mechanisms by which the samples exert the observed effects. It should be noted that, as far as we know, this is the first work where the antimicrobial properties of *P. viridis*, *M. hostilis* and the commercial mixture were studied. It should also be noted that, in most studies where the biological properties of these samples were analyzed, extraction steps were carried out with organic solvents, whereas in the present study, water was used as solvent to mimic Ayahuasca beverages.

## Figures and Tables

**Table 1:** Total phenolic compounds and flavonoids content (mean ± standard deviation).

Samples	Phenolic Compounds (mg GAE/g Sample)	Flavonoids (mg QE/g Sample)
Commercial mixture	364.67 ± 7.66	10.29 ± 0.68
*P. viridis*	210.67 ± 7.27	8.76 ± 0.94
*B. caapi*	114.67 ± 2.44	3.91 ± 0.28
*M. hostilis*	376.80 ± 15.84	14.63 ± 1.36
*P. harmala*	78.27 ± 5.75	25.92 ± 2.56
*P. viridis + B. caapi*	150.60 ± 10.47	9.81 ± 0.67
*P. viridis + P. harmala*	132.13 ± 3.84	19.93 ± 0.95
*M. hostilis + B. caapi*	327.47 ± 9.94	6.85 ± 0.23
*M. hostilis + P. harmala*	196.13 ± 1.67	16.25 ± 0.97

GAE—gallic acid equivalents; QE—quercetin equivalents.

**Table 2:** Concentration of phenolic compounds (µg/g) in the methanol extracts from decoctions samples.

Compound	Commercial Mixture	*P. viridis*	*B. caapi*	*M. hostilis*	*P. harmala*
**Hydroxybenzoic acids**					
protocatechuic acid	417.1	272.8	122.6	109.2	4.8
4-hydroxybenzoic acid	39.6	53.2	N.D.	4.3	<LOQ
gentisic acid	<LOQ	N.D.	<LOQ	N.D.	2.8
salicylic acid	1.5	<LOQ	9.1	0.25	3.8
**Hydroxycinnamic acids**					
chlorogenic acid	N.D.	42.1	N.D.	N.D.	N.D.
neochlorogenic acid	N.D.	6.0	N.D.	N.D.	N.D.
**Flavonoids-flavanols**					
(+)-catechin	240.4	10.7	465.4	1.35	<LOQ
(-)-epicatechin	570.5	22.6	1112.0	4.6	<LOQ
quercetin-3-*O*-galactoside	7.1 *	3.0 *	N.D.	N.D.	N.D.
quercetin-3-*O*-glucoside			14.4	N.D.	N.D.
quercetin-3-*O*-rutinoside	18.7	47.4	6.3	N.D.	<LOQ
kaempferol-3-*O*-glucoside	N.D.	N.D.	<LOQ	N.D.	N.D.
kaempferol-3-*O*-rutinoside	N.D.	26.6	N.D.	N.D.	N.D.
**Flavonoids-dihydrochalcone**					
phlorizin	<LOQ	N.D.	1.0	<LOQ	N.D.

N.D.—not detected; LOQ—limit of quantitation; LOQ—0.006 µg/g (salicylic acid; quercetin-3-O-rutinoside); 0.010 µg/g (4-hydroxybenzoic acid; gentisic acid, (−)-epicatechin); 0.018 µg/g (kaempferol-3-*O*-rutinoside); 0.034 µg/g (phlorizin); * mixture of both metabolites.

**Table 3:** Antioxidant properties of the samples (mean ± standard deviation).

Samples		DPPH Free Radical Scavenging Assay			β-Carotene Bleaching Test
		IC_50_ (mg/L)	AAI	Antioxidant Activity	IC_50_ (mg/L)
Commercial mixture		5.65 ± 1.05	6.36 ± 0.13	Very Strong	224.15 ± 31.72
*P. viridis*		18.89 ± 3.01	1.90 ± 0.13	Strong	225.51 ± 49.60
*B. caapi*		26.71 ± 3.77	1.51 ± 0.10	Strong	1497.88 ± 148.52
*M. hostilis*		7.17 ± 0.84	4.40 ± 0.13	Very Strong	243.35 ± 0.61
*P. harmala*		211.67 ± 20.41	0.14 ± 0.03	Poor	2713.16 ± 649.32
*P. viridis + B. caapi*		29.71 ± 3.46	1.98 ± 0.13	Strong	237.52 ± 31.94
*P. viridis + P. harmala*		29.74 ± 5.62	1.29 ± 0.04	Strong	322.55 ± 12.10
*M. hostilis + B. caapi*		7.74 ± 1.25	6.20 ± 1.39	Very Strong	1069.59 ± 65.74
*M. hostilis + P. harmala*		12.14 ± 2.42	3.27 ± 0.15	Very Strong	257.83 ± 50.28
Positive controls	gallic acid	2.23 ± 0.02	22.77 ± 0.25	Very Strong	-
	BHT	-	-	-	29.33 ± 0.34

IC_50_—half maximal inhibitory concentration; AAI—antioxidant activity index; BHT—butylated hydroxytoluene.

**Table 4:** Anti-inflammatory activity results (mean ± standard deviation).

Sample		Anti-Inflammatory Activity—IC_50_ (mg/L)
Commercial mixture		30.89 ± 1.24
*P. viridis*		168.92 ± 25.49
*B. caapi*		163.75 ± 23.84
*M. hostilis*		43.84 ± 0.46
*P. harmala*		37.38 ± 2.78
*P. viridis + B. caapi*		117.88 ± 15.91
*P. viridis + P. harmala*		N.D.
*M. hostilis + B. caapi*		N.D.
*M. hostilis + P. harmala*		19.06 ± 0.87
Positive control	Acetylsalicylic acid	0.80 ± 0.09

N.D.: Not detected; IC_50_—half maximal inhibitory concentration.

**Table 5:** Diameter of inhibition zones (mm) in disc diffusion assay (mean ± standard deviation).

Samples (3 mg/disc)		Strains							
		*S. aureus* ATCC 25923	*L. monocytogenes* LMG 16779	*E. faecalis* ATCC 29212	*B. cereus* ATCC 11778	*E. coli* ATCC 25922	*S.* Typhimurium ATCC 13311	*P. aeruginosa* ATCC 27853	*A. baumannii* LMG 1025
*P. viridis*		19.85 ± 0.28	8.19 ± 1.68	9.20 ± 1.70	14.06 ± 2.06	12.04 ± 2.49	8.84 ± 0.88	12.73 ± 1.65	12.65 ± 1.32
*B. caapi*		17.52 ± 0.69	11.04 ± 1.75	9.83 ± 2.14	17.86 ± 0.16	13.56 ± 1.51	11.01 ± 0.29	8.14 ± 1.61	17.53 ± 0.33
*P. harmala*		14.34 ± 2.03	14.54 ± 1.87	9.86 ± 0.04	16.95 ± 0.37	18.19 ± 1.52	16.93 ± 0.88	9.09 ± 0.43	17.81 ± 0.34
*M. hostilis*		19.93 ± 3.41	11.18 ± 0.91	10.13 ± 1.33	14.68 ± 1.44	13.19 ± 1.30	11.58 ± 0.54	14.59 ± 0.25	14.66 ± 0.70
Commercial mixture		14.68 ± 1.58	6.49 ± 0.85	6.00 ± 0.00	13.92 ± 1.17	7.48 ± 1.33	6.56 ± 0.98	9.73 ± 0.30	11.8 ± 0.69
*P. viridis + B. caapi*		13.69 ± 2.87	6.00 ± 0.00	6.00 ± 0.00	11.55 ± 1.77	6.00 ± 0.00	6.00 ± 0.00	10.34 ± 1.00	11.04 ± 0.77
*P. viridis + P. harmala*		13.27 ± 1.73	6. 68 ± 1.18	6.00 ± 0.00	12.52 ± 2.27	11.56 ± 0.83	11.36 ± 2.81	10.57 ± 0.90	13.87 ± 0.36
*M. hostilis + B. caapi*		20.39 ± 0.94	10.82 ± 1.57	9.09 ± 0.89	14.12 ± 2.14	11.00 ± 1.84	11.71 ± 1.44	14.57 ± 1.18	15.09 ± 1.22
*M. hostilis + P. harmala*		17.94 ± 1.29	7.44 ± 1.26	8.55 ± 1.12	14.18 ± 1.77	12.68 ± 1.10	12.12 ± 0.37	12.29 ± 2.23	15.06 ± 0.53
Controls	DMSO (15 µL/disc)	6.00 ± 0.00	6.00 ± 0.00	6.00 ± 0.00	6.00 ± 0.00	6.00 ± 0.00	6.00 ± 0.00	6.00 ± 0.00	6.00 ± 0.00
	Tetracycline (30 µg/disc)	30.25 ± 0.50	18.25 ± 0.60	25.20 ± 0.58	30.00 ± 0.82	23.25 ± 0.50	28.45 ± 0.52	11.50 ± 0.58	25.63 ± 0.25

DMSO—dimethyl sulfoxide.

**Table 6:** Minimum inhibitory concentration (MIC) values (mg/mL) of samples (modal values).

Samples		Strains							
		*S. aureus* ATCC 25923	*L. monocytogenes* LMG 16779	*E. faecalis* ATCC 29212	*B. cereus* ATCC 11778	*E. coli* ATCC 25922	*S.* Typhimurium ATCC 13311	*P. aeruginosa* ATCC 27853	*A. baumannii* LMG 1025
*P. viridis*		5	10	10	0.156	>10	>10	>10	5
*B. caapi*		1.25	2.5	10	0.313	2.5	2.5	5	0.625
*P. harmala*		1.25	5	5	0.625	1.25	2.5	5	0.625
*M. hostilis*		2.5	10	2.5	0.156	5	>10	>10	0.625
Commercial mixture		2.5	5	2.5	0.313	10	10	10	2.5
*P. viridis + B. caapi*		>10	10	>10	2.5	10	10	10	2.5
*P. viridis + P. harmala*		>10	>10	10	2.5	10	>10	10	5
*M. hostilis + B. caapi*		>10	>10	>10	2.5	5	10	5	1.25
*M. hostilis + P. harmala*		5	10	5	5	2.5	10	2.5	0.625
Controls	DMSO (%)	>20	>20	>20	>20	>20	>20	>20	>20
	Tetracycline (µg/mL)	0.06	0.06	0.06	0.06	0.06	0.24	0.06	0.24

DMSO—dimethyl sulfoxide.

**Table 7:** Anti-quorum sensing activity of the samples (mean ± standard deviation).

Samples (3 mg/disc)	Diameters of Inhibition of the Violacein Pigment Production (mm)
Commercial mixture	4.31 ± 0.23
*P. viridis*	2.44 ± 0.05
*B. caapi*	13.26 ± 1.50
*M. hostilis*	0.00 ± 0.00
*P. harmala*	13.16 ± 0.04
*P. viridis + B. caapi*	3.60 ± 0.66
*P. viridis + P. harmala*	3.49 ± 0.35
*M. hostilis + B. caapi*	3.11 ± 0.28
*M. hostilis + P. harmala*	9.62 ± 1.05
DMSO (15 μL/disc)	0.00 ± 0.00
Resveratrol (5 μg/disc)	8.49 ± 0.20

DMSO—dimethyl sulfoxide.
